# The evolution of digital health: a global, Latin American, and Brazilian bibliometric analysis

**DOI:** 10.3389/fdgth.2025.1582719

**Published:** 2025-05-30

**Authors:** Maria Eulália Vinadé Chagas, Gabriela de Oliveira Laguna Silva, Gabriel Ricardo Fernandes, Gabriela Tizianel Aguilar, Mariana Motta Dias da Silva, Evandro Luis Moraes, Isadora D Avila Lottici, Jerusa da Rosa de Amorim, Tiago de Abreu, Taís de Campos Moreira, Felipe Cezar Cabral

**Affiliations:** ^1^ Responsabilidade Social, Hospital Moinhos de Vento, Porto Alegre, Brazil; ^2^ Instituto de Pesquisa, Hospital Moinhos de Vento, Porto Alegre, Brazil; ^3^ Superintendência Administrativa, Hospital Moinhos de Vento, Porto Alegre, Brazil; ^4^ Gerência de Tecnologia de Informação, Hospital Moinhos de Vento, Porto Alegre, Brazil; ^5^ Gerência Médica de Saúde Digital, Hospital Moinhos de Vento, Porto Alegre, Brazil

**Keywords:** bibliometric analysis, digital health, Latin America, Brazil, telemedicine

## Abstract

**Introduction:**

Digital health provides remote healthcare assistance, contributing to reducing inequalities in access to services. For its widespread adoption, it is essential to disseminate successful models implemented in countries with developed digital health networks, so that they can be adapted and replicated in developing regions. The dissemination of scientific studies on the topic, combining digital health activities within various contexts with scientific research, is crucial for promoting significant advancements in the understanding and application of these technologies. This study aims to conduct a comprehensive bibliometric analysis of global scientific production in digital health from 2019 to 2024, with special attention to Latin America and Brazil.

**Methods:**

A bibliometric analysis was conducted with searches in PubMed, Scopus, and Web of Science. The analysis used the Bibliometrix package in RStudio, and the data were filtered for the global dimension, Latin American countries, and Brazil. The authorship analysis was restricted to publications with at least one Brazilian author and was carried out through a manual check of each record. The protocol was registered on the Open Science Framework platform under the number 10.17605/OSF.IO/43WQ5.

**Results:**

A total of 51,723 publications were included in the global dimension, 2,410 in Latin America, and 1,317 in the Brazilian analysis. The number of publications increased from 2019 to 2021. In the global scenario, the United States led scientific production in digital health, whereas Brazil led in Latin America.

**Conclusion:**

Digital health has expanded exponentially, consolidating itself as a strategic pillar in healthcare systems. Investments in international collaborations that encourage knowledge exchange, strengthen research networks, and drive scientific publications are essential. These partnerships are crucial for adapting digital tools to different socioeconomic contexts and ensuring equitable care for the population.

## 1 Introduction

Although telemedicine has been an increasingly relevant topic over the past few decades, it was not until the COVID-19 pandemic that its potential and persistent challenges to its global and equitable implementation became truly evident (1). As a broad concept, digital health has established itself as a strategic tool for mitigating inequalities in healthcare access, thereby driving the agenda of the United Nations' third Sustainable Development Goal, which aims to ensure universal health coverage (2). However, obstacles such as system integration, financial limitations, cultural and social differences, and challenges in technological adoption remain significant. These challenges highlight the need for innovative and collaborative solutions, as well as the enhancement of remote care modalities, to make digital health accessible and effective in diverse contexts (3).

In Latin America, for example, there is a significant limitation regarding data interoperability. Some countries face substantial weaknesses in their ability to store and manage health information (4, 5). Moreover, the development of digital competencies among healthcare professionals is still in its early stages, and public policies focused on digital health are, in many cases, either non-existent or poorly structured (6, 7). Another critical issue is the absence of culturally relevant training programs aimed at the population, which often distrusts the available digital tools (8). In the specific case of Brazil, challenges related to interoperability among the various systems used by the Unified Health System (SUS) are evident. The lack of standardization in patient data collection undermines continuity of care, especially for those who move through different levels of healthcare. Furthermore, the systems currently in use often fail to address the actual needs of public health management, hindering the understanding of population demands and limiting the health system's responsiveness, as observed during public health crises (9, 10).

The progress of digital health is particularly relevant in promoting remote healthcare models, which have the potential to mitigate inequalities and provide more accessible and equitable care (3, 11–14). Scientific publications play a crucial role in disseminating knowledge, especially by sharing tested models from strong economies, enabling their adaptation to less developed contexts (1). Identifying recent innovations and analyzing the implementation of digital health projects are essential for recognizing both progress and gaps in the field. Bibliometric analyses are valuable in this context, as they organize and assess scientific literature through techniques that explore academic credibility and impact (15). Rapid digital transformation in healthcare has significantly altered the interaction between professionals and patients, demanding robust references to support new studies (16).

Furthermore, bibliometric analyses assist the scientific community in identifying new innovation focuses and updating perspectives on the implementation of digital health projects. This approach highlights what has already been achieved, what can still be improved, and how advancement 2017s can be applied in different scenarios (17). Bibliometric analyses provide valuable insights into the thematic evolution of digital health by mapping trends, strategic collaborations, and key academic and institutional actors (18, 19). These analyses show how publications have responded to global and local challenges while identifying areas of greater academic impact, such as emerging technologies and digital health solutions (20). By connecting quantitative data with specific contexts, these tools help outline new research directions, strengthen collaborative networks, and support strategic decisions that promote equitable advancements in digital health.

Thus, this study aims to conduct a comprehensive bibliometric analysis of global scientific production in digital health from 2019 to 2024, with special attention to Latin America and Brazil, the authors' countries of origin. Mapping publication trends, identifying the most prominent countries and institutions, and exploring established collaborations and research gaps, could significantly contribute to the advancement of digital health research. This effort seeks to foster scientific and practical development in underserved regions and promote equity in healthcare access.

## 2 Methods

### 2.1 Registration

The protocol for this bibliometric analysis has been registered on the Open Science Framework (OSF) platform under the identifier DOI: 10.17605/OSF.IO/43WQ5.

### 2.2 Inclusion and exclusion criteria

Articles published between January 2019 and November 2024 were included if their scope included topics related to digital health across different fields of research. There were no restrictions regarding the language, country of origin, or institutional affiliation of the authors, acknowledging the predominance of English in scientific communication. Similarly, no initial filters were applied based on country of origin or institutional affiliation, aiming to capture the global dynamics of digital health research. Subsequently, specific analyses were conducted for Latin America and Brazil, the authors’ countries of origin, during the data segmentation phase, enabling a more detailed exploration of these regions.

Publications such as editorials, comments, letters, interviews, and news articles, as well as articles classified as Retracted Publications or documents marked as Item Withdrawals, were excluded, along with those containing a Published Erratum.

Works categorized as Practice Guidelines, Consensus Development Conferences, or publications combining different classifications—such as Journal Articles associated with Published Erratum, Retracted Publication, Book Chapter, Review, or Practice Guideline—were also excluded from the study. Finally, articles without authors or with anonymous authorship and those lacking an abstract were excluded, as the absence of this information compromises the initial assessment of relevance and content.

### 2.3 Databases and search strategy

The databases used were PubMed, Scopus, and Web of Science. The search strategy was adapted to the syntax of each database: in PubMed, the terms used were (Telemedicine[mh] OR Telemedicine[tiab] OR Digital Health[tiab]); in Web of Science, the search was performed with ALL = (Telemedicine OR “Tele medicine” OR “Tele-medicine” OR “Digital health”); and in Scopus, the query was TITLE-ABS-KEY (Telemedicine OR “Digital Health”). In all cases, the Boolean operator OR was applied to combine related terms.

Searches were conducted on November 13, 2024, across all data sources, applying a filter to limit the results to publications between January 1, 2019, and the search date.

### 2.4 Data extraction and study processing

**Figure 1:**
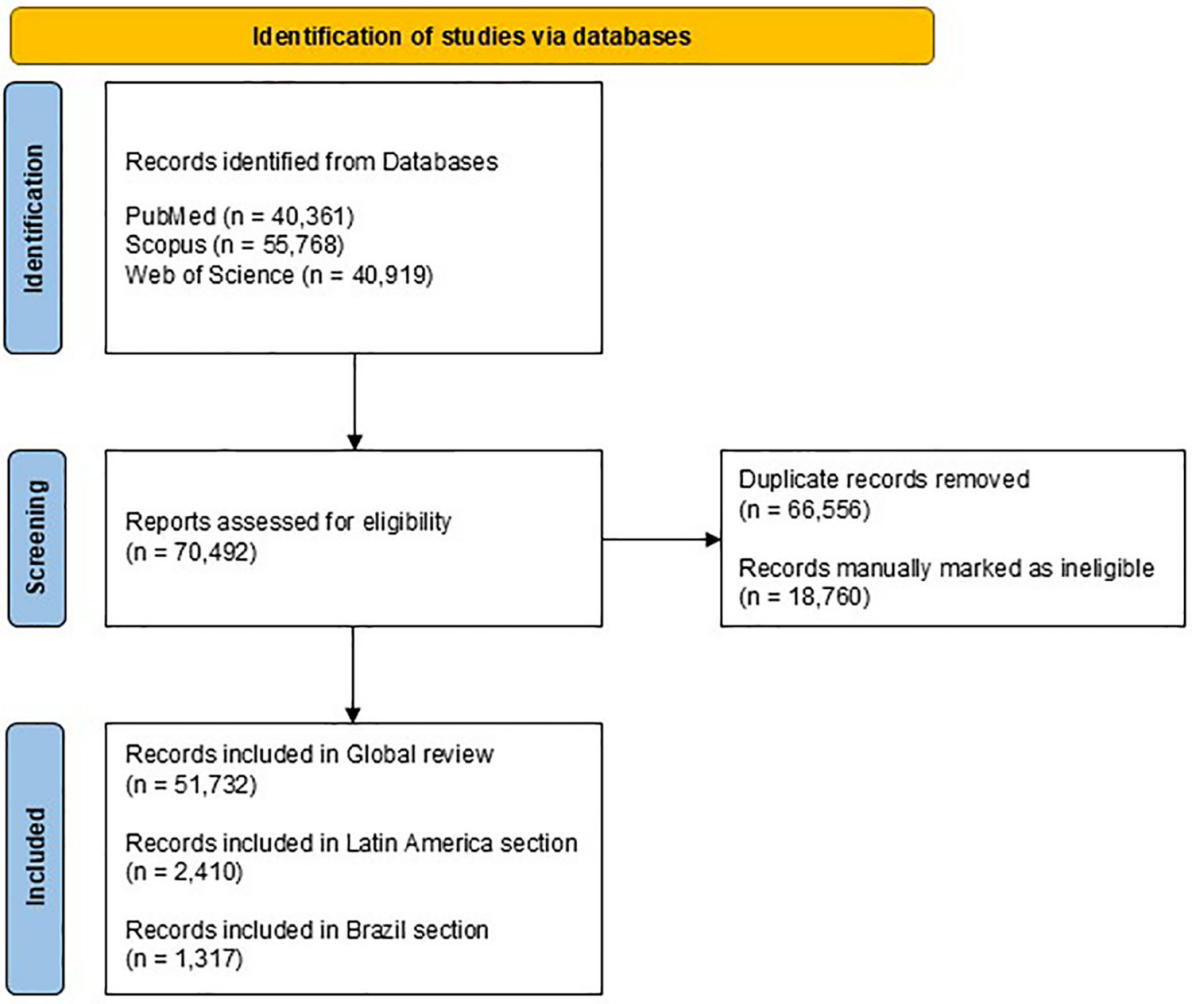
Study identification flowchart.

The search results were exported in the following formats: .nbib for PubMed, text file for Web of Science, and .csv for Scopus. The information was consolidated using the open-source software RStudio (version 4.0.0) (21). The database was consolidated into an Excel file containing bibliographic information such as authors' names, institutional affiliations, article titles, journal names, keywords, year of publication, and DOI. Duplicates were removed, and the exclusion criteria mentioned were rigorously applied to ensure the consistency of the analyzed data (Figure 1).

### 2.5 Data cleaning and organization

The data were imported into the RStudio environment using the *readxl* and *dplyr* packages for initial processing and cleaning. To handle the diversity of languages in publications, ISO 8859-1 encoding was applied to convert special characters, ensuring data integrity.

Concerning Latin America, the results included data where at least one author's affiliation was linked to one of the 20 countries in the region. For the analysis of publications from Brazil, the same criterion was applied, considering publications with at least one author affiliated with Brazilian institutions.

Author names were standardized to ensure database consistency and to facilitate the identification of Brazil's most prolific authors. This process included removing diacritics, extra spaces, and numerical inconsistencies, using functions such as *str_remove_all* and *str_trim* from the *stringr* package in R. In cases where multiple authors were listed in a single cell, their names were reorganized into individual rows, maintaining their association with the corresponding publications. Institutional affiliations were also standardized by harmonizing text entries, removing diacritics, correcting spacing, and unifying minor textual variations, to improve consistency in institution names across records.

The processed data were subsequently exported to Excel, where they were grouped on the basis of the adjusted names of the authors, and similar names were consolidated into a standardized version using semi-automated clustering based on the Jaro-Winkler similarity metric (stringdist package). This step was essential to avoid duplications caused by abbreviations or different citation formats, ensuring an accurate count of publications. To guarantee the final accuracy of the database, manual verification of each record was performed during the analysis. This verification was conducted exclusively for articles by Brazilian authors, allowing for greater precision in identifying authors and ensuring that all publications were correctly accounted for.

Finally, the detailed analysis included identifying the most studied topics, recurring keywords, Brazil's most influential authors, the temporal evolution of scientific outputs, the most relevant journals, and research networks.

### 2.6 Data analysis

The results were analyzed using the Bibliometrix package in RStudio, with graphs and tables generated through its graphical interface, biblioshiny. These results were exported in PNG format for documentation and presentation purposes. The analysis included only information classified as *acceptable* in biblioshiny and with no more than 20% missing data across the databases. Visualizations were created to identify the most studied topics, keyword occurrences, collaborations between countries and institutions, the temporal evolution of publications, and the main journals publishing on the subject.

## 3 Results

The searches conducted in the PubMed, Scopus, and Web of Science databases returned a total of 137,048 records. After removing duplicates and applying strict exclusion criteria, 51,723 articles were considered, with 2,410 publications related to Latin America and of these, 1,317 related to Brazil (Figure 1).

**Figure 2:**
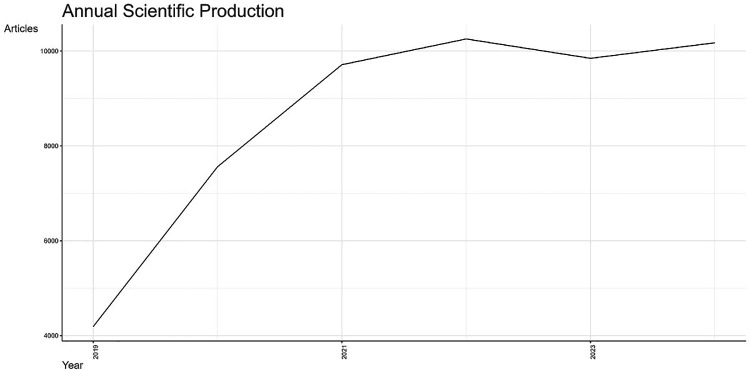
The annual evolution of digital health scientific production between 2019 and 2024 worldwide.

Global scientific production analysis revealed a significant increase in the number of publications between 2019 and 2021, which was likely associated with the impact of the COVID-19 pandemic. In 2022, there was a decline in the number of publications, but the quantity remained high compared with that in 2019 and 2020. In 2023, a recovery in scientific production was observed, with a slightly higher number than in 2022, highlighting the consolidation and growth of digital health as a globally relevant research field (Figure 2). The publications from Latin America and Brazil reflect the same trend as global scientific production (Supplementary Figures S1, S2). The analysis incorporated global, Latin American, and Brazilian data with the aim of enabling comparisons and identifying growth opportunities across different contexts.

**Figure 3:**
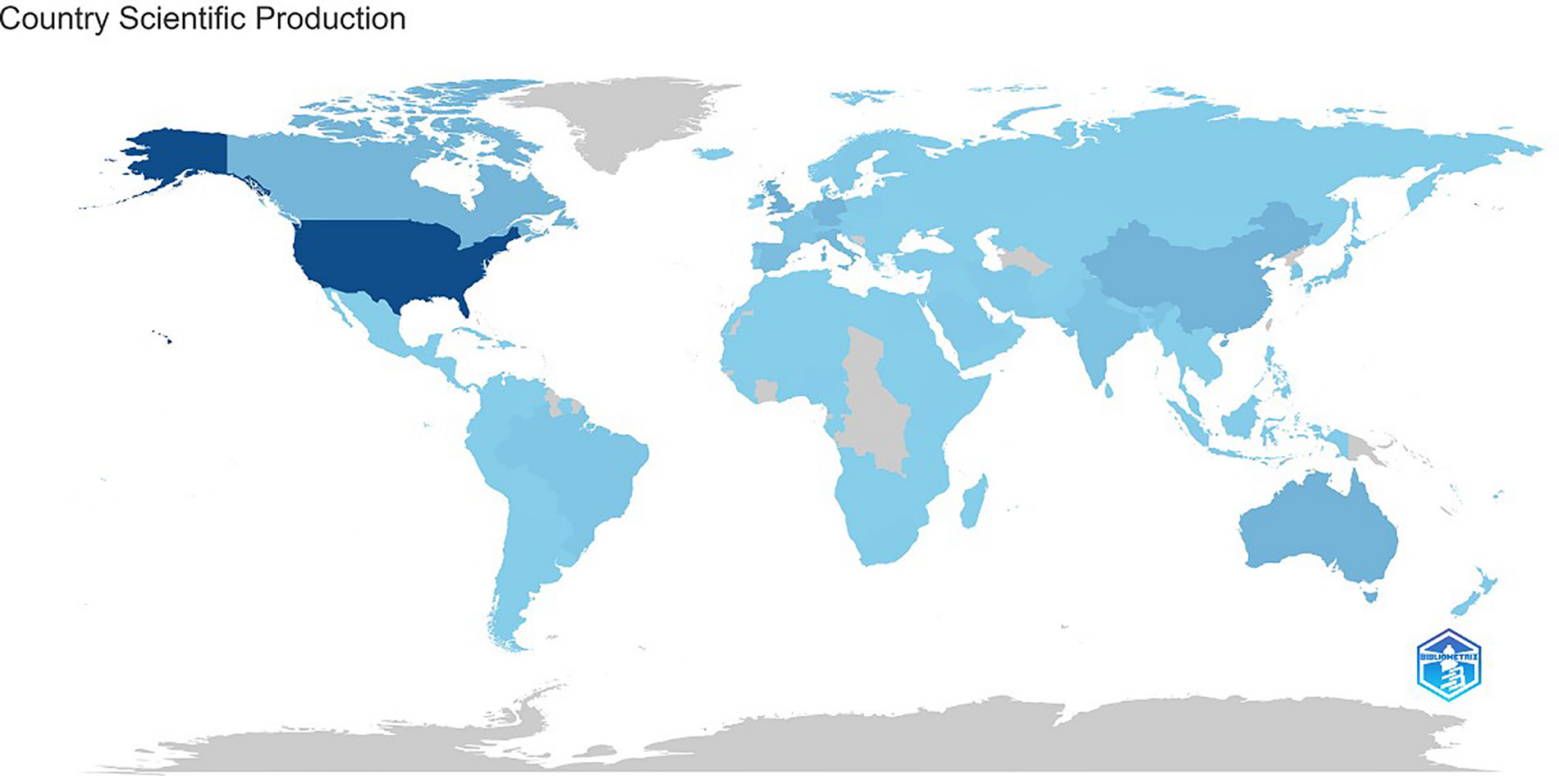
World map showing the geographical distribution of digital health scientific production between 2019 and 2024. Darker shades indicate a higher number of publications.

Regarding scientific production by country, the United States led the digital health field, followed by the United Kingdom, Australia, and China (Figure 3). Brazil ranks 12th in scientific production worldwide. However, the country holds a prominent position in Latin America, accounting for 54.6% of its publications, followed by Chile and Colombia.

**Figure 4:**
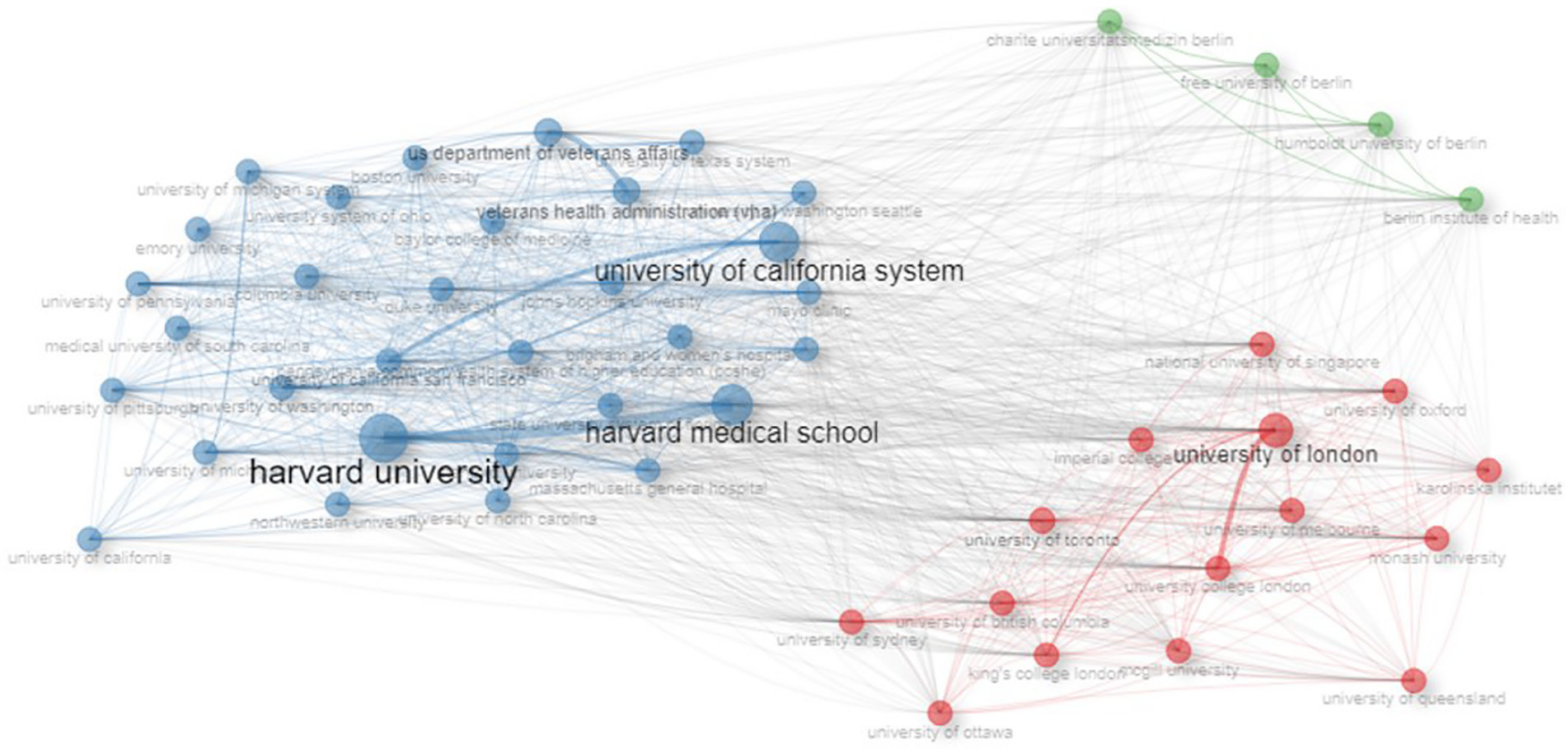
Three main clusters were identified: led by Harvard University (blue), University of London (red), and German universities (green). The density of connections reflects global interaction among the institutions.

Global institutional collaborations were grouped into three main clusters, highlighting the centrality and influence of leading institutions in the digital health field. The blue cluster, led by Harvard University, in collaboration with Harvard Medical School and the University of California System, showed the highest centrality, establishing itself as the most influential core in global collaborations. The red cluster, centered around the University of London, brought together institutions from the United Kingdom and other countries, whereas the green cluster was primarily composed of German universities, reflecting strong regional integration in Europe. Among the three clusters, the blue cluster stands out not only for its centrality but also for its global influence, reflecting the strategic role of institutions such as Harvard University in fostering scientific partnerships (Figure 4).

To complete the analysis, a word cloud and thematic evolution analysis were conducted using the keyword-plus data. The word cloud analysis was quite similar across the global, Latin American, and Brazilian analyses, with the most prominent keywords being *telemedicine*, *human*/*humans*, *female*/*male*, and terms related to the COVID-19 pandemic (Supplementary Figures S3–S5). The thematic evolution analysis was divided into two periods: 2019–2021 and 2022–2024. The analyses revealed considerable differences among the groups, although the keyword telemedicine remained a central and common term across all regions, highlighting its global and regional importance. The global analysis revealed a more general approach, including terms such as *care* (Supplementary Figure S6). In Latin America, the prominence of mHealth and the emergence of the term artificial intelligence were notable, indicating growing interest in technologies and data-driven health innovations (Supplementary Figure S7). In Brazil, studies covered a broader range of topics between 2019 and 2021, with an emphasis on specific clinical conditions. Between 2022 and 2024, however, there was a transition toward more comprehensive digital health approaches and the integration of the deep learning concept into the research landscape (Supplementary Figure S8).

The growing prominence of keywords such as mHealth, artificial intelligence, and deep learning in specific analyses, particularly in Latin America and Brazil, reflects an increasing focus on integrating advanced digital technologies into clinical and public health practices. This thematic evolution reinforces the ongoing trends of digital transformation in healthcare, especially toward personalized care models, remote interventions, and data-driven decision-making processes.

These findings suggest a progressive maturation of the digital health field, evolving from broader conceptual discussions to more targeted and technology-driven applications. Furthermore, the observed regional differences highlight the importance of tailoring digital health strategies to local contexts, considering the available technological infrastructure and the priorities of individual healthcare systems.

**Figure 5:**
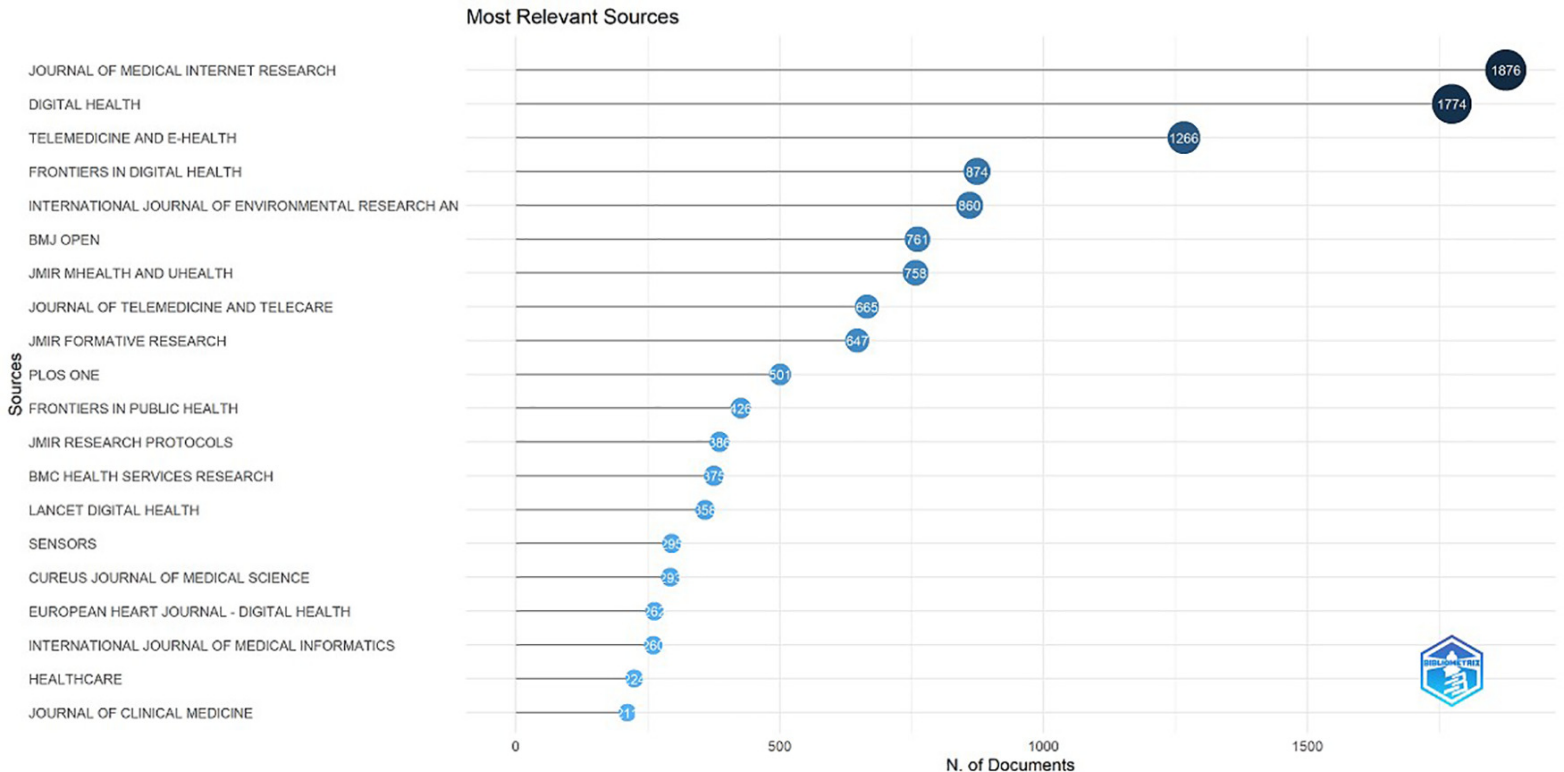
The top 20 journals with the highest number of publications based on the total number of articles published in the world from 2019 to 2024.

Figure 5 presents the rankings of the 20 most relevant sources in publications on the topic (Figure 5). In the first position is the Journal of Medical Internet Research with 1,876 publications. Among the 20 sources, 13 are related to the digital health field, whereas the other 7 are generally multidisciplinary sources.

In Latin America, Brazil stands out with the highest number of institutions in the top 20 most relevant affiliations, with the University of São Paulo (USP) leading as the most productive, followed by the Federal University of Minas Gerais (UFMG) and the Federal University of Rio Grande do Sul (UFRGS) (Supplementary Figure S9). After Brazil, Chile, Argentina, Peru, and Colombia are tied in second place, each with two institutions, and finally, Mexico has one university among the 20 most relevant affiliations in Latin America.

The Brazilian cluster highlighted patterns regarding the most studied topics, publication outlets, and the most impactful researchers in the field of digital health. The most frequent trigrams (continuous sequences of three words) found in titles of the studies were *Primary Health Care* and *Randomized Controlled Trial*, emphasizing research focused on primary healthcare and the development of robust clinical models, reflecting the pursuit of scalable and applicable solutions for the challenges of the Unified Health System (SUS), Brazil's public healthcare system (Supplementary Figure S10).

**Figure 6:**
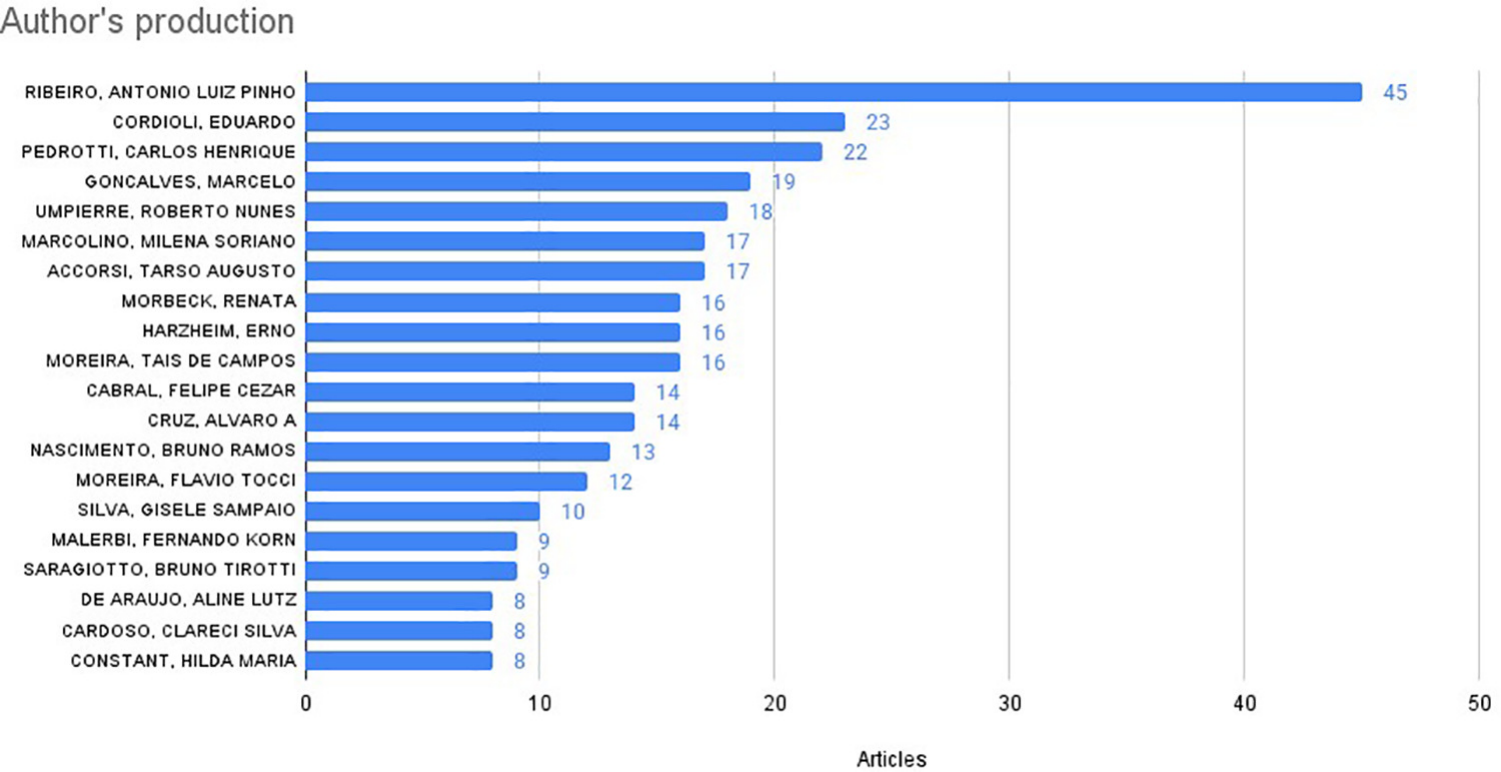
The top 20 authors with Brazilian affiliations based on the highest number of publications worldwide from 2019 to 2024.

The analysis of Brazilian affiliations highlighted researchers with more significant contributions to the field of digital health (Figure 6). These 20 authors were affiliated with 21 different institutions. Among them, the Albert Einstein Israelite Hospital had seven authors in the top 20, the Federal University of Minas Gerais (UFMG) and the Federal University of Rio Grande do Sul (UFRGS) each had four authors. The Moinhos de Vento Hospital (AHMV), the Health Technology Assessment Institute (IATS), and the Federal University of Bahia each had three affiliated authors. The remaining institutions had one affiliated author each.

## 4 Discussion

This bibliometric study provides a comprehensive overview of scientific production in the evolution of digital health between 2019 and 2024, highlighting the field's rapid growth and its global and regional dynamics. The significant increase in publications between 2019 and 2021 can be attributed to the impact of the COVID-19 pandemic, which accelerated the adoption of digital technologies to address the global health crisis (22, 23). This period represented not only an emergency response but also the strengthening of support networks and scientific collaboration, which are essential for accelerating technological innovations across various contexts (24, 25). After this accelerated growth, the publication rate slowed in 2023; however, it remained steady, signaling a phase of consolidation. This transition marks the maturation of digital health as a strategic focus in global scientific research, in alignment with international recommendations to strengthen health systems through technological innovations (23, 26). The bibliometric analysis reveals a shift from topics initially focused on emergencies, such as the implementation of telemedicine and digital surveillance, to broader and more sustainable themes, such as data governance, artificial intelligence in clinical decision-making, and equity in digital health. This evolution reflects a growing awareness of the need to integrate digital health into long-term health strategies, rather than treating it as a temporary response to crises. Emerging themes also emphasize capacity building, digital literacy, and ethical considerations, indicating a more comprehensive approach to the digital transformation of health systems. These thematic trends not only map the intellectual structure of the field but also highlight critical areas for future research and policy development, particularly in low- and middle-income regions, where digital health may face long-standing challenges related to access and efficiency.

The thematic analysis underscored this maturation, showing a shift in the topics addressed. Research focused on broader themes, reflecting conceptual development, between 2019 and 2021. From 2022 to 2024, the focus shifted to more applied areas, such as care, telemedicine, and depression, indicating a growing focus on solutions to digital health challenges (20, 25). This progress was also driven by global scientific collaborations, which play a central role in the field's development, emphasizing the importance of institutional partnerships for advancing digital health. Broad collaborative networks, evidenced in our study and led by institutions such as Harvard University and the University of London, demonstrate how strategic connections can accelerate technological innovations, address complex challenges, and promote impactful advancements in digital health (24, 25).

Countries with advanced scientific infrastructures, such as the United States, the United Kingdom, Australia, and China, continue to lead in academic production because of their ability to integrate technological resources with robust research and development strategies (20, 27). These countries stand out not only for the volume of publications but also for their ability to transform research into practical innovation, supported by well-structured funding systems, leading academic institutions, and an environment that fosters public-private sector collaborations (20, 24, 27). Nonetheless, our study also highlights the potential of emerging regions, such as Brazil, which, despite facing structural challenges, has shown significant growth in digital health scientific production.

During the analyzed period, scientific production in Latin America followed global trends, with Brazil emerging as the leading contributor, accounting for 54.6% of regional publications (Table 1). This performance enhances Brazil's strategic position, not only in regional scientific production but also in its integration into global innovation networks. This positioning reflects Brazil's ability to combine regional and international collaborations, promoting scientific initiatives that strengthen the collective impact in the field (28–30). In addition to Brazil, other Latin American nations, such as Chile, Mexico, and Argentina, have also contributed significantly, with initiatives particularly focused on areas like telemedicine. These efforts demonstrate attempts to adapt technological infrastructures to local realities, promoting solutions aligned with regional needs and highlighting Latin America's potential as a whole in advancing digital health (31).

**Table 1:** Most relevant affiliations—Latin America.

Affiliations	Country	Articles
University of São Paulo (USP)	Brazil	383
Federal University of Minas Gerais (UFMG)	Brazil	244
Federal University of Rio Grande do Sul (UFRGS)	Brazil	148
Federal University of São Paulo (Unifesp)	Brazil	147
Hospital Israelita Albert Einstein	Brazil	117
Pontificia Universidad Catolica De Chile (UC Chile)	Chile	115
Universidad De Chile	Chile	110
Italian Hospital of Buenos Aires	Argentina	89
Federal University of São Carlos (UFSCar)	Brazil	81
Universidade Federal Do Rio Grande Do Norte (UFRN)	Brazil	76
University Of Buenos Aires	Argentina	71
Universidad Peruana Cayetano Heredia	Peru	67
Universidad Nacional Mayor De San Marcos	Peru	58
Pontificia Universidad Javeriana	Colombia	53
State University of Campinas (UNICAMP)	Brazil	52
Federal University of Bahia (UFBA)	Brazil	51
Oswaldo Cruz Foundation (FIOCRUZ)	Brazil	47
Federal University of Rio de Janeiro (UFRJ)	Brazil	46
Universidad Icesi	Colombia	42
National Autonomous University of Mexico (UNAM)	Mexico	40

Leading affiliations in digital health scientific production in Latin America (2019–2024).

Regarding institutions, the University of São Paulo (USP), the Federal University of Minas Gerais (UFMG), and the Federal University of Rio Grande do Sul (UFRGS) led scientific production in Brazil. The thematic analysis revealed that these institutional efforts align with local priorities, such as primary healthcare, and initiatives focused on developing robust clinical models (28, 30). The most frequent trigrams, such as Primary Health Care and Randomized Controlled Trial, point to a strategic focus on research addressing the challenges of the Unified Health System (SUS) and seeking scalable and practical solutions to improve access and efficiency in healthcare services (31, 32). This thematic focus not only consolidates Brazil's position as a significant player in digital health but also highlights its potential as a model for other emerging economies. Aligning local priorities with global demands has emerged as a promising path for generating scalable, inclusive, and sustainable solutions.

### 4.1 Comparisons with prior work

Previous studies, such as by Tian et al. (33), explored global trends in eHealth between 2000 and 2021, emphasizing the expansion of topics like telemedicine during the pandemic. Our work, by including more recent data and expanding the scope to multiple databases, complements this analysis by outlining the thematic evolution toward more applied areas that emerged as practical priorities in addressing digital health challenges.

Furthermore, Yang et al. (34) addressed digital literacy, highlighting its importance in empowering both professionals and patients in the use of emerging technologies. Although our study did not directly analyze digital literacy, it broadens the understanding by including data on emerging economies, demonstrating how countries such as Brazil have significantly contributed to scientific production in digital health in Latin America. Brazil's strategic relevance in this context reinforces the need to integrate emerging economies into the global dialog, as observed in previous studies that showed a lack of geographical diversity in publications (33).

This perspective of including developing countries in global discussions was also illustrated in bibliometric analyses focused on the Journal of Medical Internet Research (JMIR) (20, 34, 35). These studies emphasized the need to broaden thematic and geographical diversity of publications, highlighting regional disparities and the underrepresentation of developing countries. Based on these findings, our work contributes by highlighting how Brazil and other Latin American countries have advanced in scientific production in digital health, despite facing challenges related to technological infrastructure and connectivity, particularly in remote regions. These results reinforce the importance of investing in international collaborations that promote inclusion and more effectively integrate local and global efforts, expanding the impact of technological innovations in digital health.

### 4.2 Limitations

The authorship analysis was impacted by the lack of uniformity in the recording of researchers' names in scientific journals, an inherent limitation of the software used, Bibliometrix, which only counts exact matches without unifying similar records or correcting inconsistencies. For data from Brazil, this issue was mitigated through detailed manual verification, ensuring greater accuracy in the normalization of names. Despite these efforts, inconsistencies in name standardization at the global and Latin American levels may have impacted the identification of prolific authors and the formation of collaboration networks. Although the use of persistent identifiers, such as the ORCID iD, was considered, their still inconsistent presence in the retrieved records limited their systematic application.

Additionally, the terminology in digital health is characterized by its constant evolution, presenting challenges in capturing terms that describe concepts, technologies, and practices. Regional differences, less frequent terms, emerging nomenclature, or variations in terminology associated with digital health may not have been captured, potentially leading to an incomplete representation of global and regional scientific production. These limitations may have directly influenced the identification of the most productive authors and the configuration of collaboration networks, particularly in the global and Latin American contexts. The fragmentation of authorship records may have led to an underestimation of the number of publications attributed to certain researchers and to the dispersion of collaboration nodes in the mapped networks. Additionally, we acknowledge that the rapid evolution of digital health terminology may have resulted in the omission or underrepresentation of emerging terms, potentially affecting the analysis of thematic evolution. While these factors do not compromise the overall trends observed, they should be taken into account when interpreting the breadth and dynamics of the findings.

These limitations highlight the need for improvements in bibliometric analysis tools and methodologies that incorporate more robust data clustering strategies, capable of keeping pace with rapid transformations in the digital health field. This would provide a more accurate analysis of global and regional collaborations, ensuring greater representativeness and comprehensiveness in data interpretation. It is recommended that future bibliometric studies use digital identifiers to improve name disambiguation and the accuracy of collaboration mapping.

## 5 Conclusion

This study reinforces digital health as a strategic field that transcends geographical and economic barriers, promoting collaborations that connect diverse regions to global innovation networks. As the field evolves, the integration of emerging economies into the global dialog, the expansion of thematic diversity, and overcoming structural barriers will be essential to ensure that the benefits of digital innovations are widely distributed and sustainable. Prioritizing investments in international collaboration, technological infrastructure, and capacity building, as well as integrating academic research into healthcare services and national policies, is crucial to consolidating digital health as an indispensable pillar of more resilient global health systems that are better prepared for future challenges.

## Data Availability

The dataset analyzed in this review is registered on the Open Science Framework (OSF) platform under the identifier DOI: 10.17605/OSF.IO/43WQ5.
