# Supplementary material for: The evolution of digital health: a global, Latin American, and Brazilian bibliometric analysis
Source: Front Digit Health. 2025 May 30;7:1582719. doi: 10.3389/fdgth.2025.1582719 (PMC12162614; doi:10.3389/fdgth.2025.1582719)
Supplement: Supplementary file 1 [file Datasheet1.pdf]

frontiers

Supplementary Material.

1 Supplementary Figures

Supplementary Figure 1 - Annual Scientific Production - Latin America

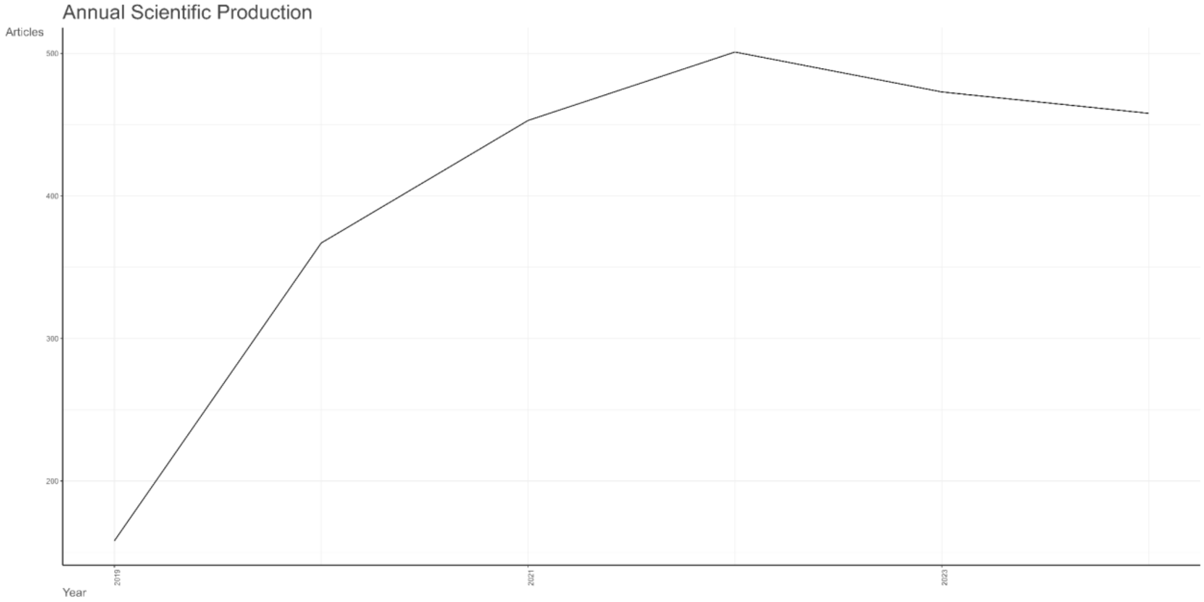

Supplementary Figure 1. The annual evolution of scientific production in digital health between 2019 and 2024 in the Latin America-focused analysis.

Supplementary Material

Supplementary Figure 2 - Annual Scientific Production – Brazil

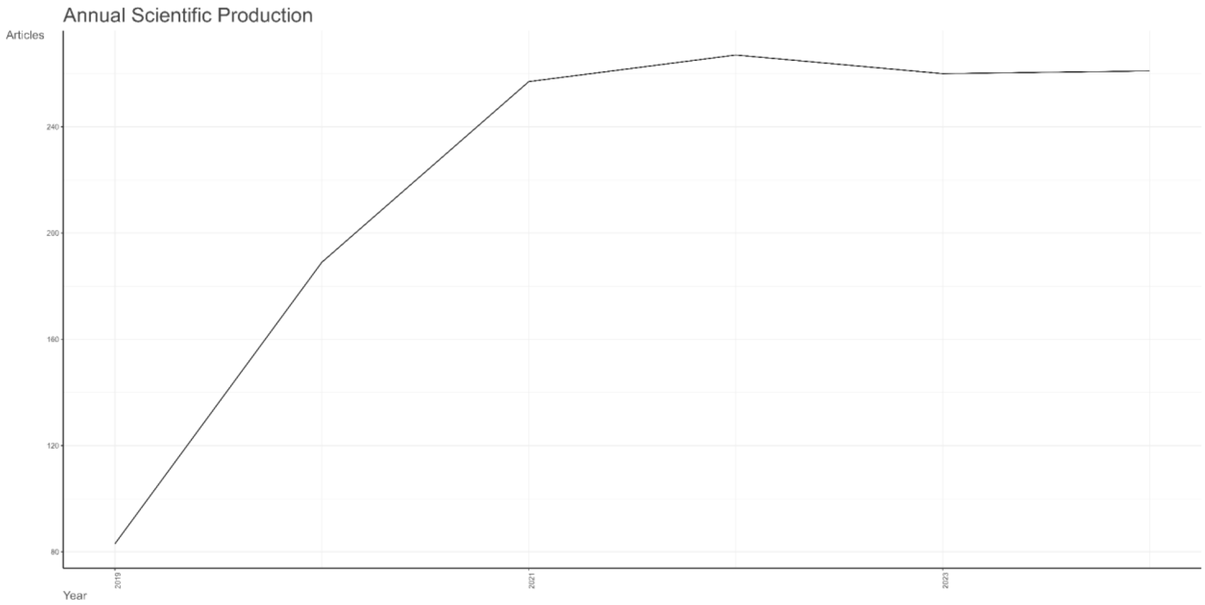

Supplementary Figure 2. The annual evolution of scientific production in digital health between 2019 and 2024 in the Brazil-focused analysis.

Supplementary Figure 3 - Word Cloud – Global

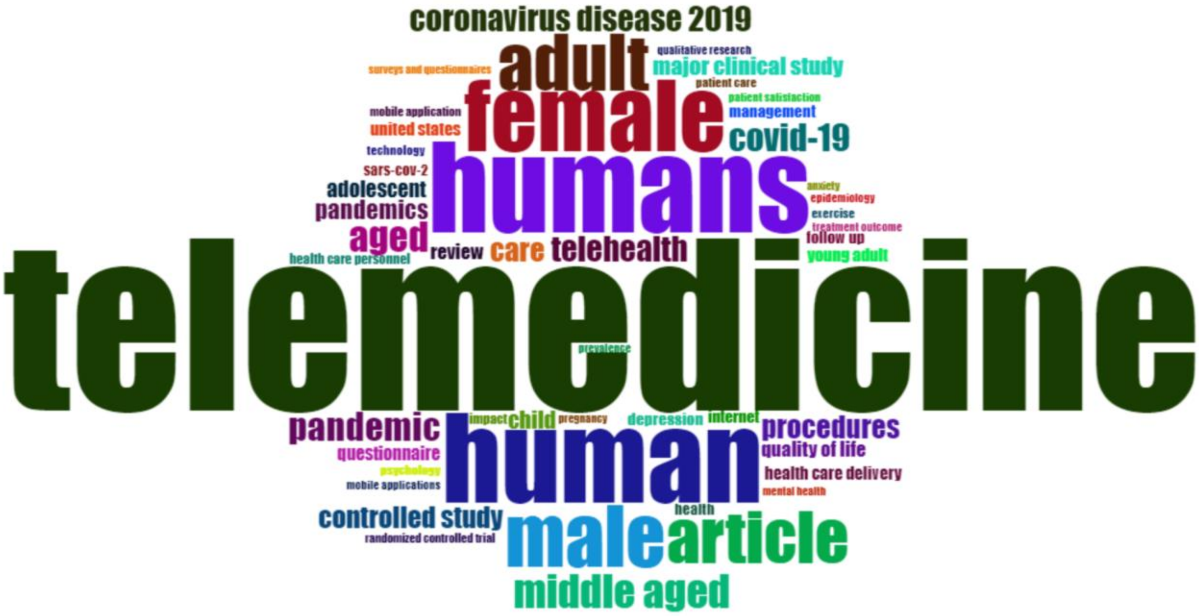

Supplementary Figure 3. Word cloud of the 50 most frequently occurring keyword-plus in the global-focused analysis.

Supplementary Material

Supplementary Figure 4 - Word Cloud - Latin America

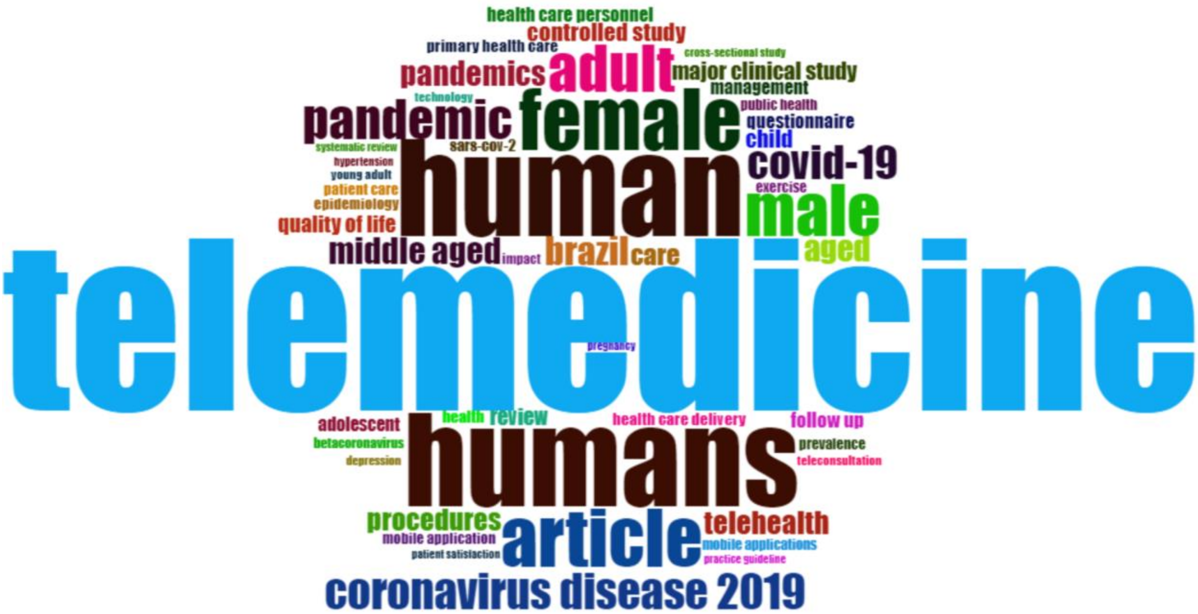

Supplementary Figure 4. Word cloud of the 50 most frequently occurring keyword-plus in the Latin America-focused analysis.

Supplementary Figure 5 – Word Cloud – Brazil

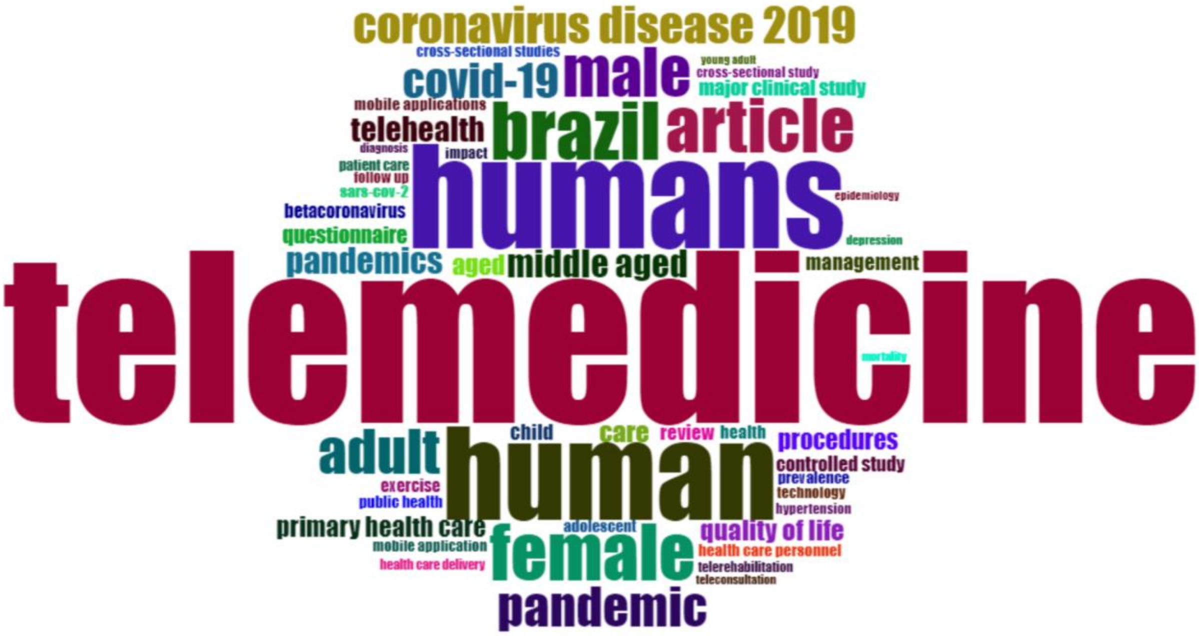

Supplementary Figure 5. Word cloud of the 50 most frequently occurring keyword-plus in the Brazil-focused analysis.

Supplementary Figure 6 - Thematic Evolution Global

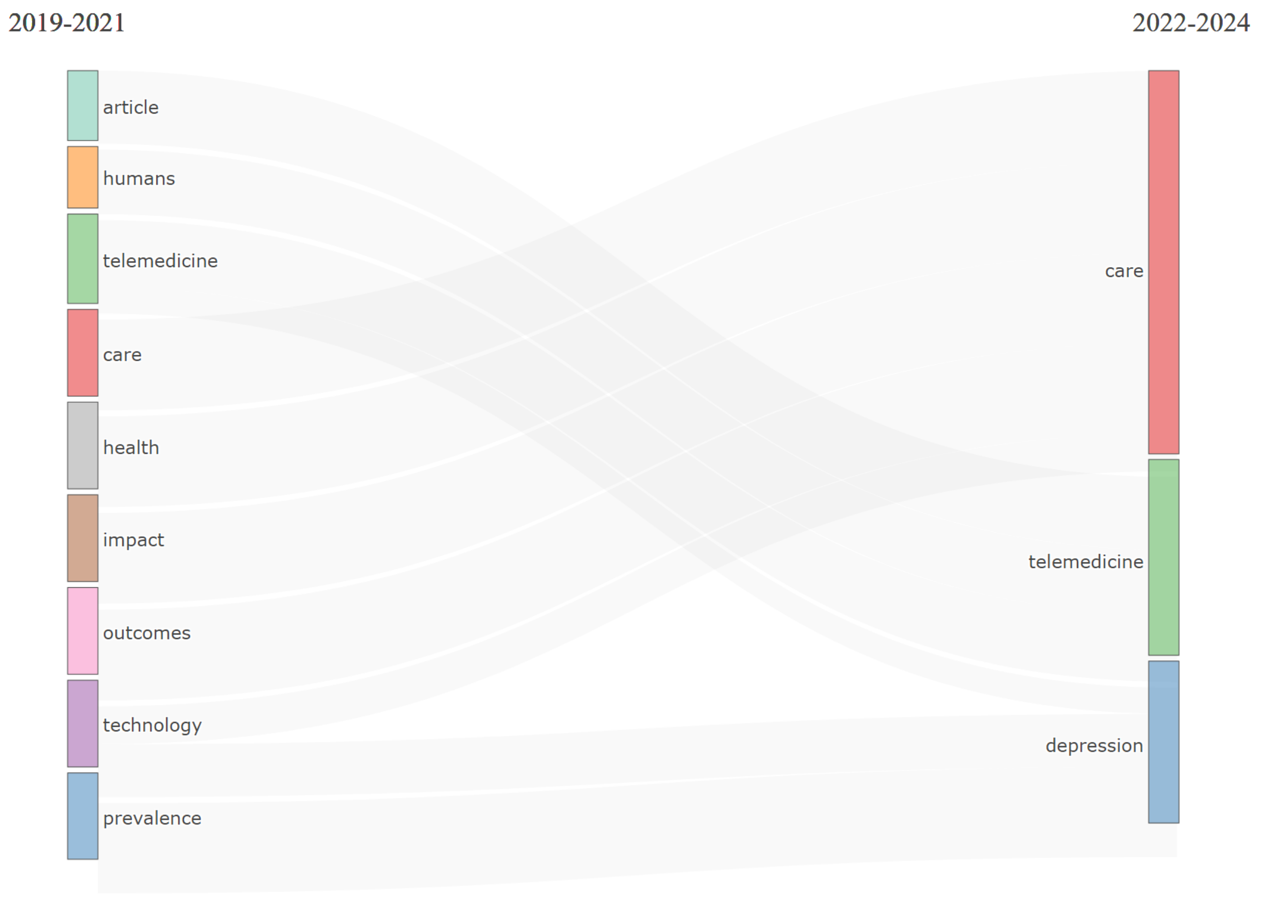

Supplementary Figure 6. Thematic evolution based on the keywords-plus from the analyzed articles in the global-focused analysis. The first column, for the period 2019-2021, presents a subdivision into 9 topics, which in the second period (2022-2024) condense into only three topics. The connecting lines illustrate how themes evolved and merged over time.

Supplementary Figure 7 - Thematic Evolution Latin America

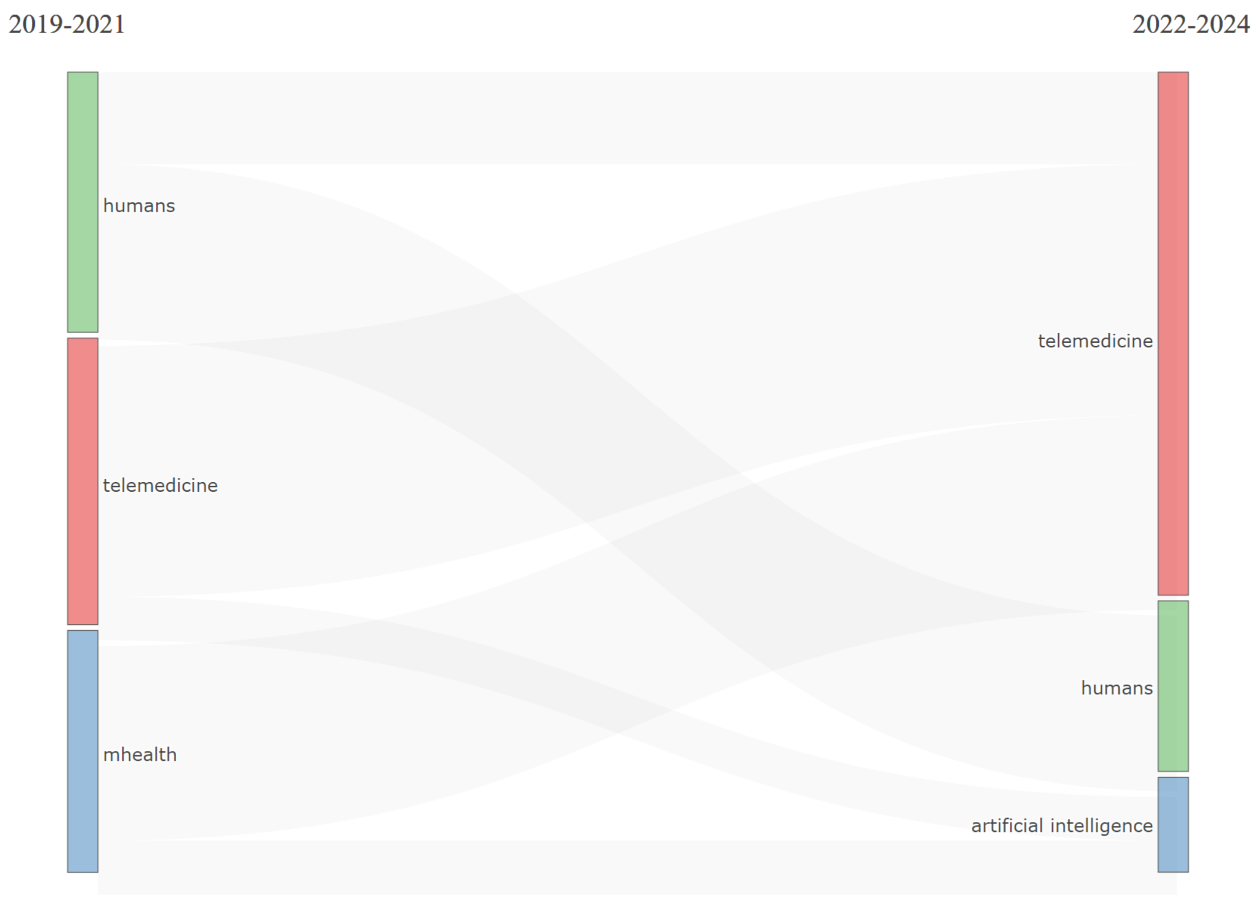

Supplementary Figure 7. Thematic evolution based on the keywords-plus from the analyzed articles in the Latin America-focused analysis. The first column, for the period 2019-2021, presents a subdivision into 3 topics, which in the second period (2022-2024) also condense into three topics. The connecting lines reflect the transformation of the topics over time.

Supplementary Material

Supplementary Figure 8 - Thematic Evolution Brazil

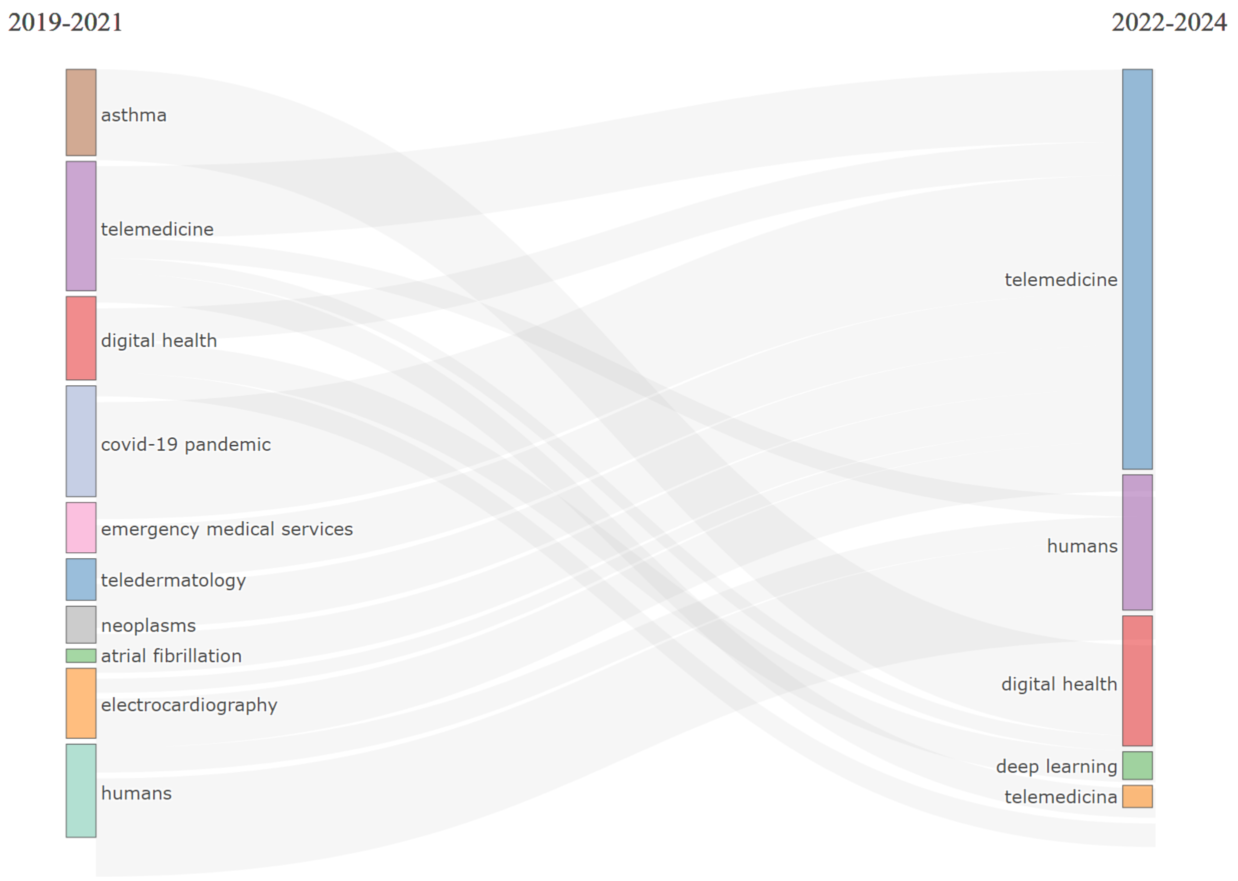

Supplementary Figure 8. Thematic evolution based on the keywords-plus from the analyzed articles in the Brazil-focused analysis. The first column, for the period 2019-2021, presents a subdivision into 10 distinct thematic clusters, which in the second period (2022-2024) condense into only five broader topics. The connecting lines illustrate how themes evolved and merged over time.

Supplementary Figure 9 - Most Relevant Affiliations Brazil

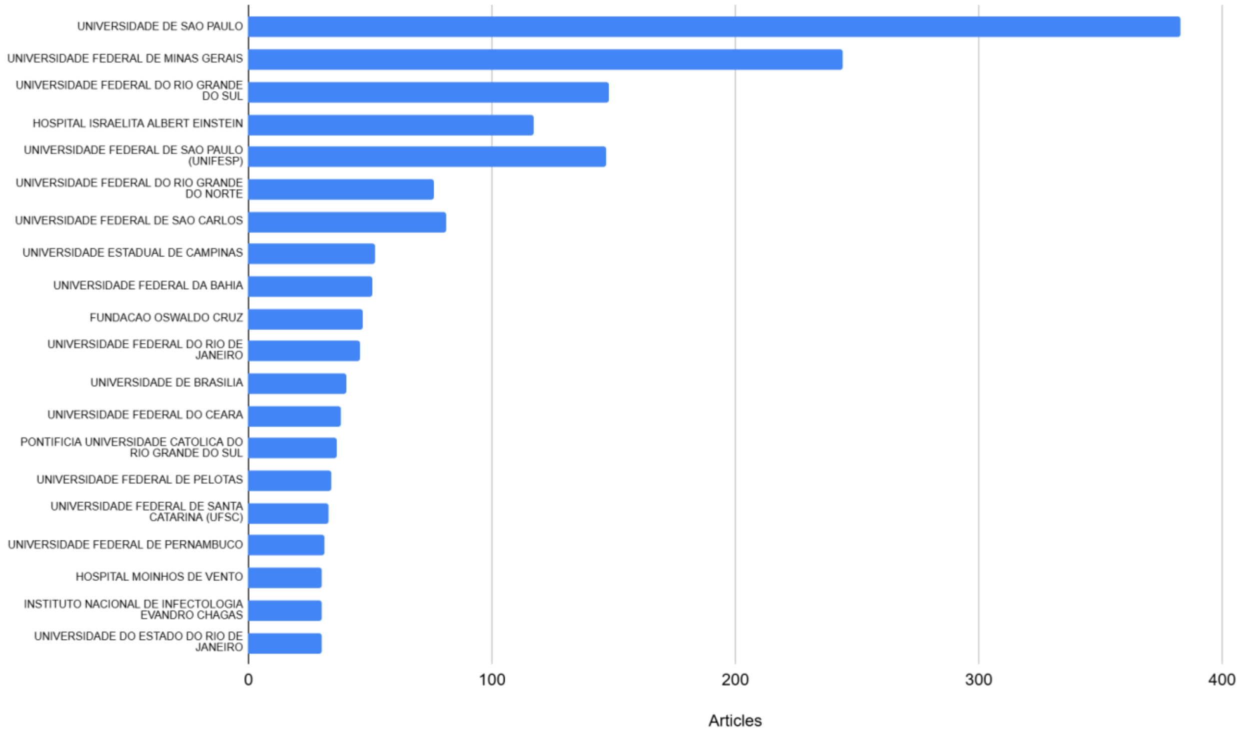

Supplementary Figure 9. Top 20 Brazilian affiliations with the highest number of publications from 2019 to 2024, based on the total number of articles published.

Supplementary Figure 10 - Word Cloud Trigrams – Brazil

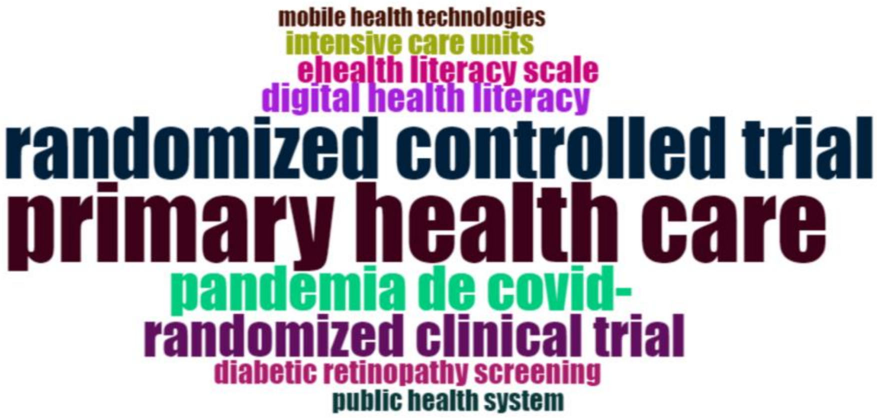

Supplementary Figure 10. Word cloud of the 10 most frequently used trigrams in study titles from Brazil. The terms are predominantly related to themes such as primary care, public health, and research methodology. Trigrams represent the possible combinations in three lines.
